# Latent KSHV Infected Endothelial Cells Are Glutamine Addicted and Require Glutaminolysis for Survival

**DOI:** 10.1371/journal.ppat.1005052

**Published:** 2015-07-21

**Authors:** Erica L. Sanchez, Patrick A. Carroll, Angel B. Thalhofer, Michael Lagunoff

**Affiliations:** 1 Molecular and Cellular Biology Program, University of Washington, Seattle, Washington, United States of America; 2 Department of Microbiology, University of Washington, Seattle, Washington, United States of America; 3 Fred Hutchinson Cancer Research Center, Seattle, Washington, United States of America; University of North Carolina at Chapel Hill, UNITED STATES

## Abstract

Kaposi’s Sarcoma-associated Herpesvirus (KSHV) is the etiologic agent of Kaposi’s Sarcoma (KS). KSHV establishes a predominantly latent infection in the main KS tumor cell type, the spindle cell, which is of endothelial cell origin. KSHV requires the induction of multiple metabolic pathways, including glycolysis and fatty acid synthesis, for the survival of latently infected endothelial cells. Here we demonstrate that latent KSHV infection leads to increased levels of intracellular glutamine and enhanced glutamine uptake. Depletion of glutamine from the culture media leads to a significant increase in apoptotic cell death in latently infected endothelial cells, but not in their mock-infected counterparts. In cancer cells, glutamine is often required for glutaminolysis to provide intermediates for the tri-carboxylic acid (TCA) cycle and support for the production of biosynthetic and bioenergetic precursors. In the absence of glutamine, the TCA cycle intermediates alpha-ketoglutarate (αKG) and pyruvate prevent the death of latently infected cells. Targeted drug inhibition of glutaminolysis also induces increased cell death in latently infected cells. KSHV infection of endothelial cells induces protein expression of the glutamine transporter, SLC1A5. Chemical inhibition of SLC1A5, or knockdown by siRNA, leads to similar cell death rates as glutamine deprivation and, similarly, can be rescued by αKG. KSHV also induces expression of the heterodimeric transcription factors c-Myc-Max and related heterodimer MondoA-Mlx. Knockdown of MondoA inhibits expression of both Mlx and SLC1A5 and induces a significant increase in cell death of only cells latently infected with KSHV, again, fully rescued by the supplementation of αKG. Therefore, during latent infection of endothelial cells, KSHV activates and requires the Myc/MondoA-network to upregulate the glutamine transporter, SLC1A5, leading to increased glutamine uptake for glutaminolysis. These findings expand our understanding of the required metabolic pathways that are activated during latent KSHV infection of endothelial cells, and demonstrate a novel role for the extended Myc-regulatory network, specifically MondoA, during latent KSHV infection.

## Author Summary

KSHV is the etiologic agent of KS, the most common tumor of AIDS patients worldwide. Currently, there are no therapeutics available to directly treat latent KSHV infection. This study reveals that latent KSHV infection induces endothelial cells to become glutamine addicted, similarly to cancer cells. Extracellular glutamine is required to feed the TCA cycle through glutaminolysis, a process called anaplerosis. KSHV induces protein expression of the glutamine transporter SLC1A5 and SLC1A5 expression is required for the survival of latently infected cells. KSHV also induces the expression of the proto-oncogene Myc and its binding partner Max, as well as, the nutrient-sensing transcription factor, MondoA and its binding partner Mlx. MondoA regulates SLC1A5 and glutaminolysis during latent KSHV infection, and its expression is required for the survival of latently infected endothelial cells. These studies show that glutaminolysis and a single glutamine transporter, under the regulation of MondoA, are required for the survival of latently infected cells, providing novel druggable targets for latently infected endothelial cells. This work supports that a cancer-like metabolic signature is established by latent KSHV infection, opening the door to further therapeutic targeting specifically of KSHV latently infected cells.

## Introduction

Kaposi’s Sarcoma-associated Herpesvirus (KSHV) is a human γ-herpesvirus and the etiologic agent of several malignancies, including two B-cell lymphomas, primary effusion lymphoma (PEL) and Multicentric Castleman’s Disease (MCD), as well as Kaposi’s Sarcoma (KS), an angioproliferative tumor[1, 2]. KS is the most common tumor of AIDS patients worldwide and also commonly occurs in non-AIDS patients in central Africa and the Mediterranean[2–4]. KS is a highly vascularized tumor comprised predominantly of spindle cells of endothelial origin. In both KS spindle cells and endothelial cells in culture, KSHV establishes a primarily latent infection, with only a small percentage of the tumor cells undergoing lytic replication[5, 6].

How KSHV alters endothelial cells to lead to cancer is still an open question. Previous work from our lab and others has demonstrated that KSHV, similarly to cancer cells, induces several major metabolic pathways. These alterations in cellular metabolism are imperative to the survival of cells latently infected with KSHV[7–9]. During latent KSHV infection, glucose uptake is induced and lactate production is significantly increased[7]. This switch to aerobic glycolysis is characteristic of the Warburg effect, a hallmark of cancer cell metabolism[10]. Interestingly, KSHV-infected endothelial cells require the Warburg effect for their survival, as latently infected endothelial cells are extremely sensitive to drug inhibition of glycolysis[7]. Recent evidence supports that the viral miRNAs expressed during latency are sufficient for the induction of the Warburg effect in KSHV-infected cells[11].

Our lab has also shown that KSHV induces the production of lipids via fatty acid synthesis (FAS) during latent infection[8]. Over half of the long-chain fatty acids detected in our metabolomics screen were elevated following latent KSHV infection. Lipid droplet organelles were also increased by latent KSHV infection of endothelial cells, evidence of increased fatty acid synthesis. Inhibition of FAS leads to apoptosis of KSHV-infected cells, which was rescued with supplementation of palmitate, a downstream metabolic intermediate of FAS. These data indicated that downstream intermediates of FAS are required for endothelial cell survival during latent infection. Induction of both glycolysis and FAS are also required in primary effusion lymphoma cells where KSHV is present[9].

Both the Warburg effect and increased FAS are metabolic signatures found in most cancer cells[12]. In these cells, glucose is primarily being utilized to produce lactic acid and fatty acids and is therefore diverted away from the tri-carboxylic acid (TCA) cycle. The TCA cycle metabolizes carbon to produce both bioenergetic and biosynthetic precursors. Importantly, glutamine carbon can be utilized to replenish the TCA cycle through a process termed anaplerosis[13]. Glutamine is the most abundant amino acid available to mammalian cells. Cancer cells induce glutamine uptake to support a glutamine requirement that exceeds the amount that cells can synthesize. Cancer studies have shown that transformed cells become glutamine addicted, or dependent on this exogenous glutamine and its catabolism via glutaminolysis for their survival[14, 15]. Recent evidence demonstrates that glutamine addiction in some cancers is enabled by the extended Myc network. Together, Myc-Max, with MondoA, a nutrient-sensing transcription factor, and its heterodimerization partner, the Max-like protein X (Mlx), facilitate the reprogramming of cellular metabolism in Myc-overexpressing cells [16–18].

A number of lytically replicating viruses also require glutamine for maximal viral replication[19–21]. Previous studies have shown that poliomyelitis virus and human cytomegalovirus depend on both glucose and glutamine for efficient virus replication[19, 21]. Interestingly, during vaccinia virus infection, glucose is completely dispensable for viral replication, but viral infection is reliant on glutamine for maximal virion production[20]. However, no studies have examined glutamine dependence during *de novo* KSHV infection.

We show that latent KSHV infection of endothelial cells induces glutamine uptake and that infected cells are dependent on the catabolism of glutamine for their survival. In the absence of exogenous glutamine, a significant percentage of KSHV-infected endothelial cells undergo apoptosis unless supplemented with TCA cycle intermediates such as alpha-ketoglutarate (αKG) or pyruvate. Targeted drug inhibition of glutamine uptake or glutaminolysis during latent infection recapitulates the findings from the glutamine-deprived conditions.

Additionally, we show that KSHV infection induces protein expression of c-Myc, its dimerization partner Max, MondoA, and its dimerization partner, Mlx. KSHV infection also induces protein expression of the glutamine-transporter protein SLC1A5. c-Myc coordinately with MondoA/Mlx is essential for regulation of glutaminolysis in cancer cells[16, 18] and is also necessary for the induction of SLC1A5 in KSHV-infected endothelial cells. Inhibition of MondoA or SLC1A5 induces cell death in KSHV-infected cells, but not mock-infected cells, and can be rescued with supplementation of αKG. Therefore, latent KSHV infection induces and requires glutamine uptake and subsequent glutaminolysis, regulated by MondoA and the glutamine transporter SLC1A5, for the survival of latently infected endothelial cells.

## Results

### Latent KSHV Infection of Endothelial Cells Induces Increased Glutamine Uptake

**Fig 1:**
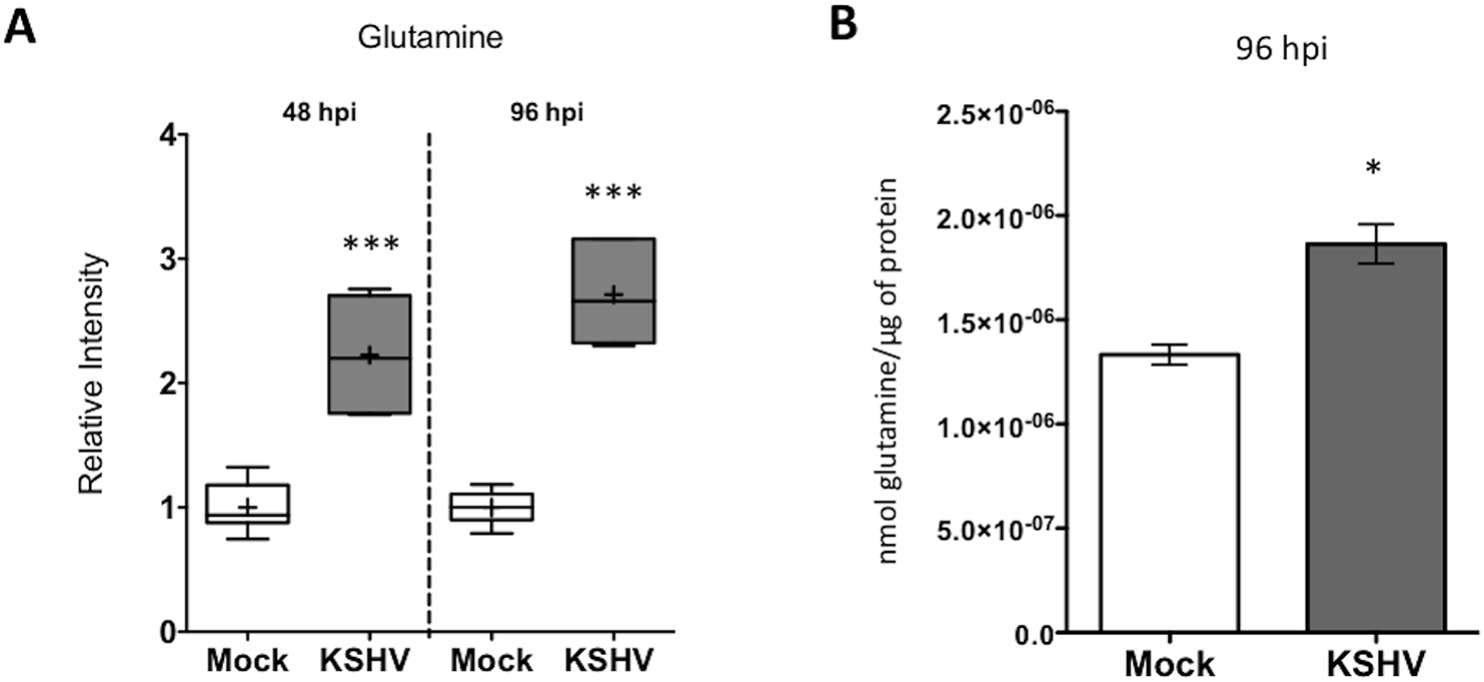
Glutamine uptake is increased by latent KSHV infection. **(A)** Intracellular glutamine levels are elevated following KSHV infection. Box and whisker plot showing relative level of intracellular glutamine determined in a metabolomics screen of Mock- (white) and KSHV-infected (grey) TIME cells at 48 and 96 hpi[8]. The average intensity of intracellular glutamine from six biological replicates is denoted by (+) sign. *P*-value < 0.0001 at both time points. **(B)** Glutamine uptake is increased during latent KSHV-infection. Ninety-six hpi, Mock- and KSHV-infected TIME cells were incubated with [^3^H]-Glutamine for 10 min, followed by intracellular quantification of radioactivity, normalized to total protein. Error bars represent the SEM of three independent experiments, *p*-value < 0.05.

A global metabolomics screen identified that glutamine levels are significantly elevated at both 48 and 96 hours post latent KSHV infection[8]. Intracellular glutamine abundance is elevated 2.2 fold at 48 hours post infection (hpi) and 2.7 fold at 96 hpi, as compared to mock-infected cells (Fig 1A). To determine if the increased levels of glutamine in infected cells was due to increased uptake during latent infection, a radiolabeled glutamine molecule, [^3^H]-Glutamine, was added to the media of mock- and KSHV-infected Tert-Immortalized Microvascular Endothelial (TIME) cells at 96 hpi. Intracellular radiolabeled glutamine levels were then determined 10 min post treatment by scintillation. Latent KSHV infection induces glutamine uptake by approximately 35% compared to mock-infected cells (Fig 1B). These data validate that elevated levels of glutamine during latent KSHV infection are a result of an increase in exogenous glutamine uptake.

### Exogenous Glutamine Is Required for the Survival of Endothelial Cells Latently Infected with KSHV

**Fig 2:**
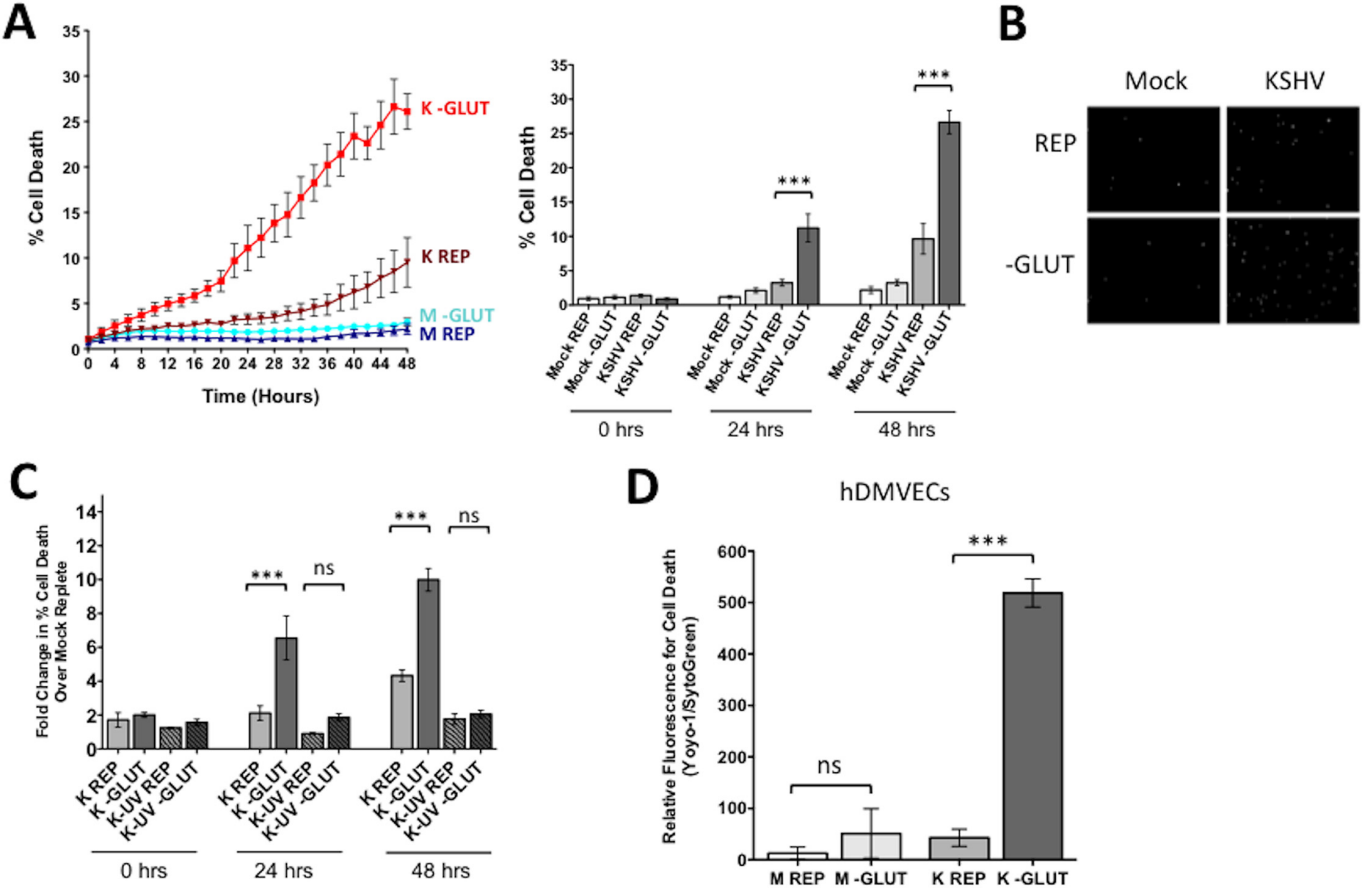
Glutamine metabolism is required for the survival of latently infected endothelial cells. **(A)** At 20 hpi, mock- or KSHV-infected TIME cells were re-seeded into 24-well plates in triplicate. Cells were treated with replete (REP) or glutamine-free (-GLUT) media containing the fluorescent dye YOYO-1 to identify dead cells or SytoGreen24 to identify total cell number. Cells were imaged every 2 hours for 48 hours on the Essen BioScience IncuCyte. The line graph displays percent cell death (Average YOYO-1 positive cells/Average SytoGreen positive cells) for every time point over 48 hours of treatment, while the bar graph shows the percent cell death at t = 0, 24, and 48 hours post treatment. Data shown represents the average of three independent experiments. Error bars are SEM, and a *p*-value < 0.0001 is represented by three asterisks. **(B)** IncuCyte microscopy images identifying dead cell nuclei (YOYO-1) for Mock- and KSHV-infected cells in replete or glutamine-free media at 48 hours post treatment (72 hpi). Essen software was used to identify cell nuclei by size and fluorescent intensity, with background subtracted. YOYO-1 positive nuclei are in white. **(C)** TIME cells were Mock-, KSHV-, or KSHV-UV (UV-irradiated) infected and prepared as described in panel A. Cells were imaged every 2 hours for 48 hours on the Essen BioScience IncuCyte. Fold increase in cell death of each sample over Mock replete at t = 0 is shown. Error bars represent SEM, *p*-value < 0.0001 and “ns” is shown where the averages are not significantly different. **(D)** Primary hDMVECs were re-seeded into 24-well plates at 48 or 72 hpi and were treated with Replete (4 mM glutamine) or glutamine-free media and 48 hours post treatment were scanned on the Typhoon 9400 variable mode imager (GE Healthcare) and analyzed with ImageJ software for relative fluorescence. Data shown represent the average of three independent experiments. Error bars are SEM. A *p*-value < 0.0001 is represented by three asterisks and “ns” is shown where the averages are not significantly different.

To determine if exogenous glutamine is a required carbon source for the survival of endothelial cells latently infected with KSHV, we quantified cell death over time in the presence or absence of exogenous glutamine. TIME cells were mock- or KSHV-infected and allowed to establish latency for 24 hours. Cells were re-seeded into 24-well plates, and overlaid with replete media, which contains 4mM glutamine, or glutamine-free media. Both treatment medias were prepared with dialyzed FBS, depleted of small molecules, including glutamine, and experiments were performed in triplicate. Average cell death over time was measured using the live-cell Essen Bioscience IncuCyte imaging system, which records both phase-contrast as well as fluorescent images over time. Dead cells were identified using the fluorescent nuclear dye YOYO-1, a cell impermeable dye that only enters cells with compromised membranes. Total cell number was determined by using SytoGreen24, a cell permeable dye that enters all cell nuclei. Percent cell death was calculated by dividing the total number of dead cells (YOYO-1 positive) by the total number of cells (SytoGreen24 positive). Cell death was monitored for 48 hours (24 hpi through 72 hpi). Fig 2A shows the average percent cell death recorded every 2 hours over 48 hours of monitoring for three biological replicate infections. The bar graph shows the average cell death at 0, 24, and 48 hours post treatment for each condition. In mock-infected cells, with replete or glutamine-deprived media, there is less than 5 percent cell death over the time monitored (Fig 2A). KSHV-infected cells in replete media have a slight increase in cell death over the time course. However, glutamine starvation of KSHV-infected cells induces a significant increase in cell death, approximately 25–30% after 48 hours of treatment (72 hpi). Microscopy images were analyzed for positive cell nuclei based on size and fluorescence intensity for both YOYO-1 and SytoGreen24. Representative images of YOYO-1 positive cells at 48 hours post treatment are shown in Fig 2B.

To ensure that glutamine addiction is not simply due to virus binding and entry, we repeated the experiments with UV-irradiated KSHV. UV-irradiated virus is able to bind and enter cells, but does not support viral gene expression. KSHV-infected cells show a 10-fold increase in cell death upon glutamine starvation, whereas UV-irradiated KSHV-infected cells show similar levels of cell death to mock-infected cells (Fig 2C). Therefore, KSHV viral gene expression is required to induce the dependence on glutamine and establish a state of glutamine addiction in endothelial cells.

To show that KSHV glutamine addiction was not limited to TIME cells, we conducted similar cell death experiments upon depletion of glutamine in mock- and KSHV-infected primary human dermal microvascular endothelial cells (1° hDMVECs). Mock- and KSHV-infected 1° hDMVECs were overlaid with glutamine depleted media at 48 or 72 hpi. Fourty-eight hours post glutamine depletion, YOYO-1 and SytoGreen24 counts were measured on a Typhoon scanner to determine relative fluorescence for cell death. These experiments reveal a significant increase in cell death only in KSHV-infected 1° hDMVECs, substantiating that KSHV infection of 1° hDMVECs also induces glutamine addiction (Fig 2D).

### Glutamine Starvation Leads to Apoptosis of KSHV Infected Endothelial Cells

**Fig 3:**
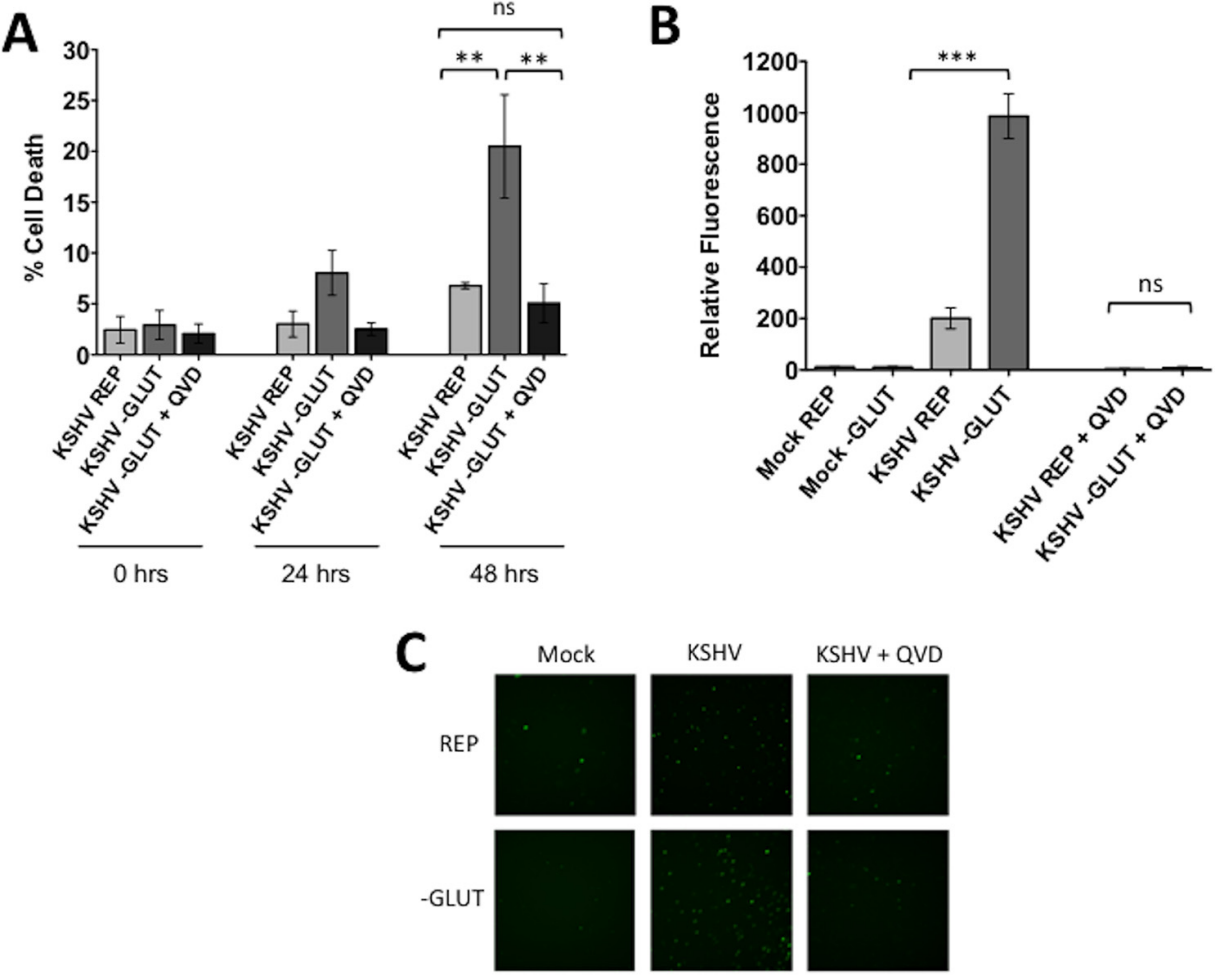
Glutamine starvation leads to apoptosis of KSHV-infected endothelial cells. At 20 hpi, Mock- or KSHV-infected TIME cells were re-seeded into 24-well plates in triplicate. Cells were treated with replete (REP) or glutamine-free (-GLUT) media in the presence or absence of 20 μM QVD (pancaspase-inhibitor) and **(A)** YOYO-1 or SytoGreen24 were added to wells to quantify dead cells and total cell numbers, respectively. Each condition was examined in triplicate, using the Essen BioSciences IncuCyte. Percent cell death was calculated (YOYO-1 positive cells/SytoGreen24 positive cells). Data represents the average of three independent experiments. Error bars represent SEM. A *p*-value < 0.01 is denoted by two asterisks and “ns” is shown for averages that are not significantly different. **(B)** The Cell Event Caspase-3/7 substrate was added to all wells. Each condition was examined in triplicate at 48 hours post treatment, and scanned for relative fluorescence on the Typhoon. Relative fluorescence shown is normalized to SytoGreen24 levels (total cell count). Data represents the average of three independent experiments. Error bars represent SEM. A *p*-value < 0.0001 is denoted by three asterisks and “ns” is shown where two averages are not significantly different. **(C)** Representative microscopy images of Caspase-3/7 samples at 48 hours post treatment (72 hpi). Live cell imaging was captured on the Cellomics ArrayScan Vti.

We have previously shown that inhibition of glycolysis and fatty acid synthesis leads to cell death via apoptosis of endothelial cells latently infected with KSHV[7, 8]. It has also been shown that glutamine deprivation leads to apoptosis of cancer cells[17]. To determine if glutamine starvation induces cell death via activation of an apoptotic pathway, we performed the previously described cell death assay using YOYO-1 and SytoGreen24 counts in the presence or absence of the pan-caspase inhibitor QVD. Upon supplementation with QVD, KSHV-infected cells deprived of glutamine are rescued from cell death (Fig 3A), indicating that cell death is due to caspase-dependent apoptosis.

To confirm that cells are dying via apoptosis, we utilized a fluorogenic Caspase-3/7 substrate, which contains the caspase cleavage site, a short four amino acid peptide (DEVD), conjugated to a nucleic acid binding dye. This cleavage site is specifically targeted by activated executioner caspases 3 and 7. When caspase 3 and/or 7 are activated during apoptosis, the DEVD site is cleaved, resulting in the release of the DNA dye, translocation to the nucleus and fluorescence. For these experiments, the Caspase-3/7 substrate was added to mock- and KSHV-infected cells in the presence or absence of glutamine at 24 hpi. After 48 hours of treatment, plates were scanned for relative fluorescence using a Typhoon 9400 variable mode imager. Caspase-3/7-mediated relative fluorescence was normalized to SytoGreen24 relative fluorescence from the same experiment. Only KSHV-infected cells starved of glutamine showed significant detection of fluorescence from the Caspase-3/7 substrate, indicating that latent infection induces Caspase 3 and/or 7 activation, which in turn results in an elevated level of DEVD cleavage and nuclear fluorescence (Fig 3B). We also included samples supplemented with QVD. These samples showed no increased fluorescence even in the absence of glutamine (Fig 3B). Representative microscopy images at 48 hours post treatment were captured with the Cellomics ArrayScan Vti (Fig 3C). Overall, these data indicate that when deprived of glutamine, KSHV-infected endothelial cells activate apoptosis in a Caspase-3/7 dependent manner.

### Glutaminolysis Is Required for KSHV Infected Endothelial Cell Survival

**Fig 4:**
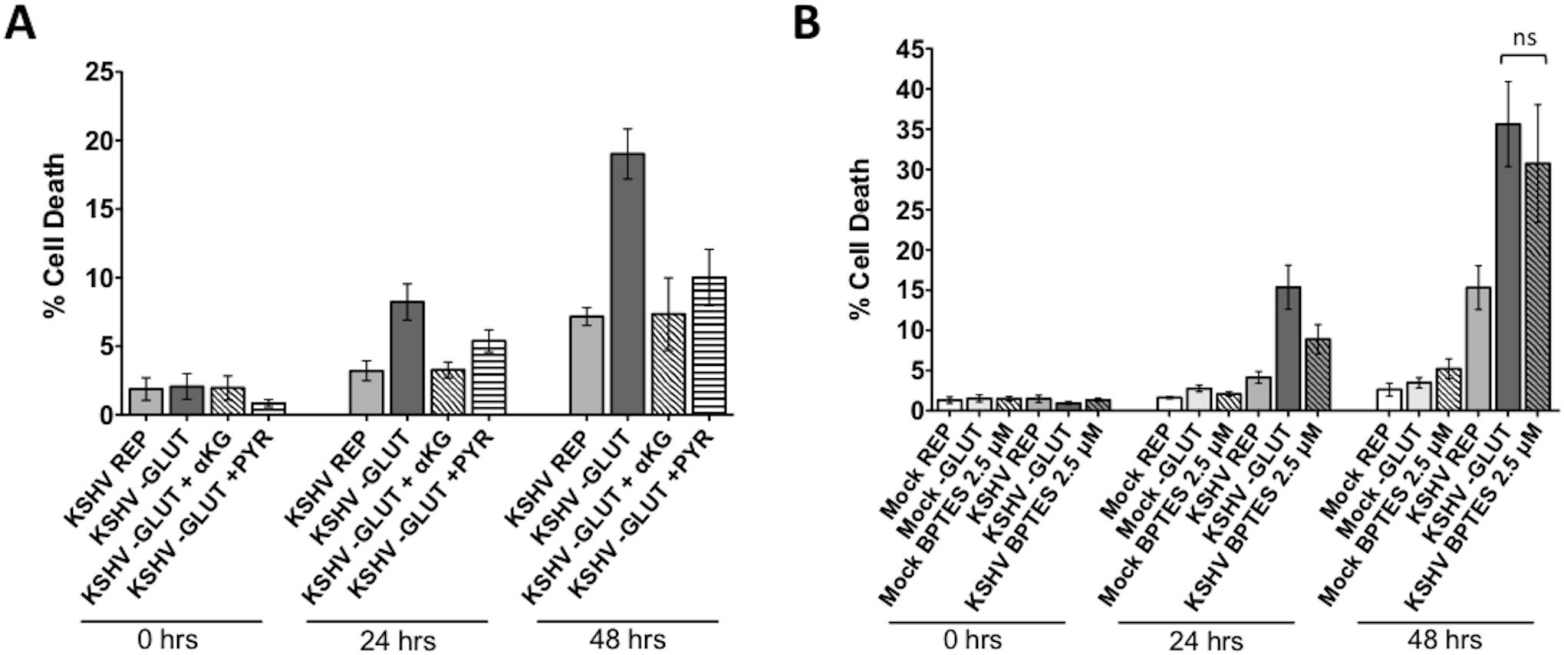
Glutamine is required for glutaminolysis in KSHV-infected endothelial cells. TCA cycle intermediates rescue KSHV-infected cells when starved of glutamine. At 20 hpi, Mock- or KSHV-infected TIME cells were re-seeded into 24-well plates in triplicate. **(A)** Cells were treated with replete (REP) or glutamine-free (-GLUT) media, supplemented with either 3.5 mM αKG or 8 mM Pyruvate and imaged on the Essen BioSciences IncuCyte for 48 hours. Percent cell death (YOYO-1 positive/SytoGree24 positive cells) is shown for t = 0, 24, and 48 hours post treatment from at least two independent experiments. Error bars represent the SEM. Mock samples showed no increase in cell death. **(B)** Treatment with 2.5 μM BPTES, a specific glutaminase inhibitor, induces cell death to the same level as glutamine-deprivation in KSHV-infected cells. Data represents the average of three independent experiments and error bars represent the SEM. The average cell death quantified in the –GLUT samples and the BPTES-treated samples are not significantly different, denoted by “ns” in the graph.

Upon entering the cell, glutamine is catabolized via glutaminolysis. Glutaminolysis consists of two consecutive deamination steps. First, glutamine is converted to glutamate by glutaminase (GLS). Second, glutamate is converted to αKG by one of three enzymes: glutamate dehydrogenase (GDH), glutamate pyruvate transaminase (GPT) or glutamate oxaloacetate transaminase (GOT)[14, 22]. At this stage, αKG can enter and replenish the TCA cycle. To determine if glutamine is required to maintain the TCA cycle in KSHV-infected cells, we added either membrane-soluble αKG or pyruvate, both of which can enter the TCA cycle, to the treatment medium of glutamine-deprived cells during latent KSHV infection. After mock- or KSHV-infection of TIME cells for 24 hours to allow the establishment latency, cells were re-seeded as before and overlaid with replete media, glutamine-free media, or glutamine-free media supplemented with either 3.5 mM αKG or 8 mM pyruvate. Supplementation with αKG completely rescues the glutamine-deprived KSHV-infected cells from cell death and supplementation with pyruvate significantly rescues cell death in the glutamine-deprived infected cells (Fig 4A). These metabolite rescue data support the model that the exogenous glutamine taken up by KSHV-infected endothelial cells is necessary to support glutaminolytic metabolism for replenishment of the TCA cycle.

BPTES is a specific inhibitor of GLS, the first enzyme of glutaminolysis. When treated with BPTES in the presence of 4mM glutamine (replete media), KSHV-infected endothelial cells died at similar levels to those deprived of glutamine, while having little effect on mock-infected cells (Fig 4B). These data recapitulate our findings with glutamine-deprived media. Taken together, these data validate that glutamine is essential for glutaminolysis in KSHV-infected cells.

### KSHV Induces Expression of the Myc/MondoA Network and Their Targets Including the Glutamine Transporter SLC1A5

**Fig 5:**
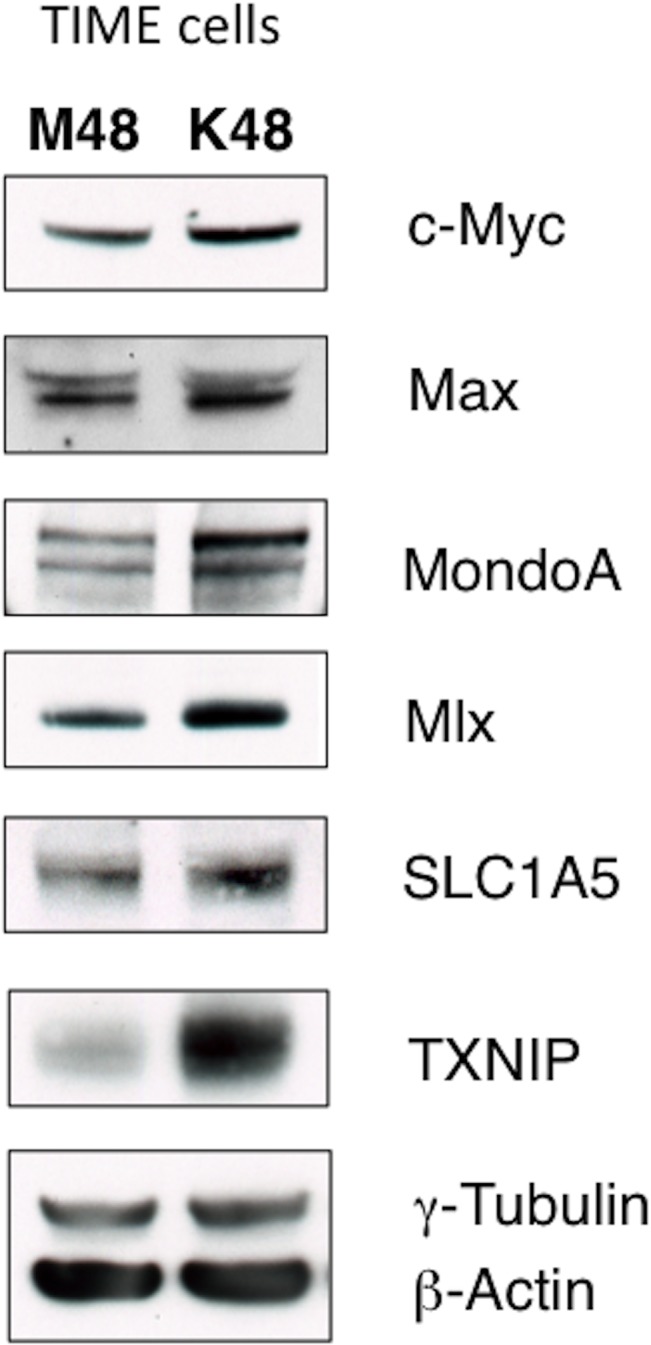
KSHV infection of endothelial cells increases protein expression of the Myc/MondoA network and downstream targets, including the glutamine transporter SLC1A5. TIME cells were Mock- or KSHV-infected and whole-cell lysates were harvested at 48 hpi. Lysates were subjected to immunoblot analysis using the indicated antibodies. Υ-tubulin and β-actin standards were included as loading controls.

Glutamine metabolism is regulated by oncogenic c-Myc in many cancer cells[16, 17, 23]. Additionally, there is evidence that c-Myc is regulated by latent KSHV infection[24, 25]. Recently, it was shown that c-Myc[26], and N-Myc[18], manipulate metabolic gene expression coordinately with the Myc-bHLHZ superfamily members MondoA, a nutrient-sensing protein, and its dimerization partner, Mlx. MondoA/Mlx or the paralogue ChREBP/Mlx constitute the “nutrient-sensing” arm of the extended Myc network[27]. Protein expression of c-Myc, Max, MondoA and Mlx are increased during latent KSHV infection of TIME cells, as determined by immunoblot analysis of whole cell lysates harvested at 48 hpi (Fig 5). Additionally, a known target of activated MondoA/Mlx, TXNIP, is upregulated at the protein level during latent KSHV infection (Fig 5).

It has been shown that Myc/MondoA controls glutamine metabolism by inducing the expression of the major glutamine transporter, SLC1A5[18]. SLC1A5 is a neutral amino acid transporter which localizes to the cellular membrane, and is known to primarily import glutamine[28]. SLC1A5 is upregulated in many cancer cells [16, 18, 28]. There is a small, but reproducible, increase in SLC1A5 protein in TIME cells latently infected with KSHV when whole cell lysates are compared by immunoblot analysis at 48 hpi (Fig 5). Together, these data suggest that latent KSHV infection induces changes to the Max/Mlx-regulation network consistent with coordinate regulation of metabolism, including glutamine uptake through SLC1A5.

### The Glutamine Transporter SLC1A5 Is Required for Survival of Endothelial Cells Latently Infected with KSHV

**Fig 6:**
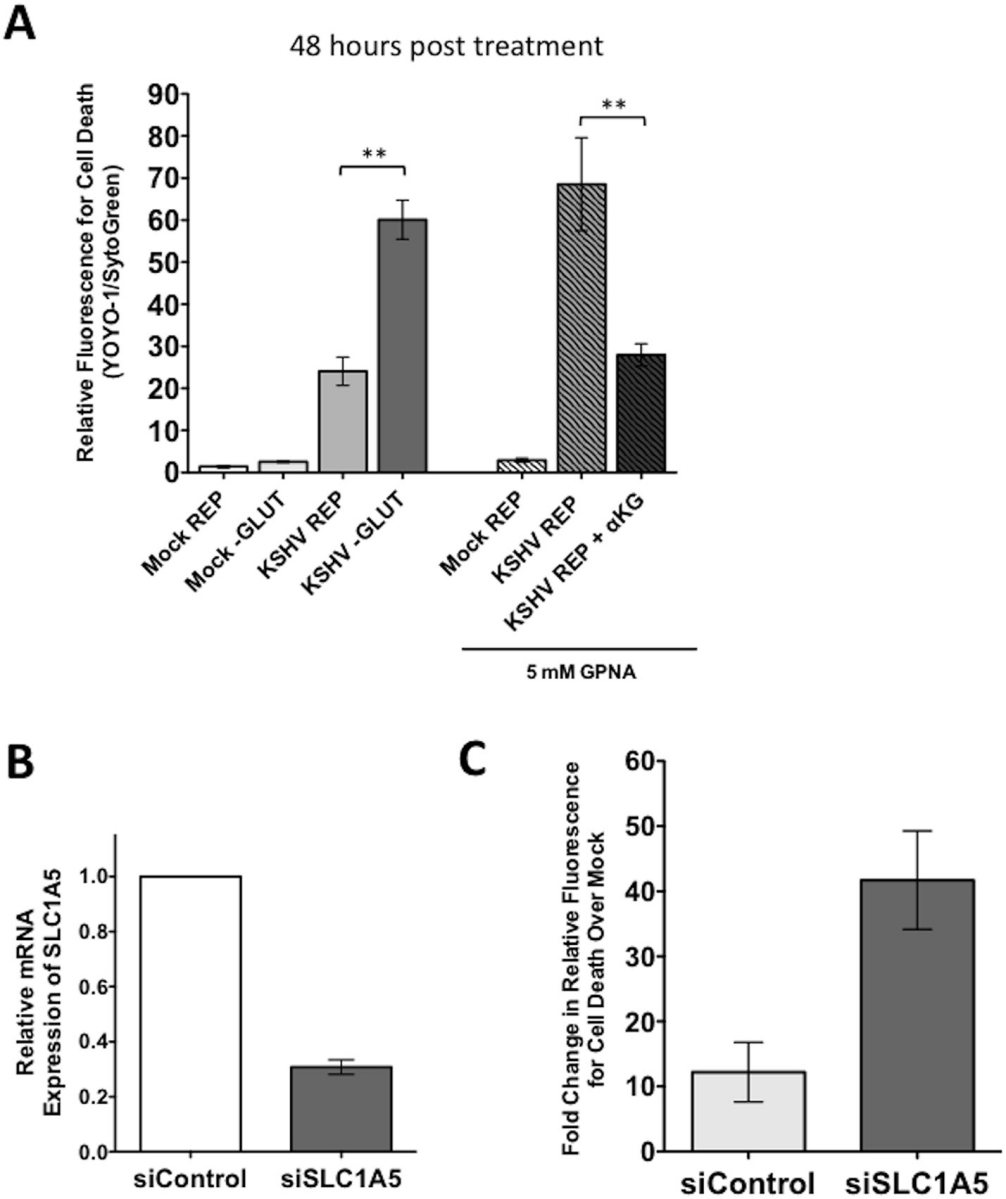
Endothelial cells latently infected with KSHV require the glutamine transporter SLC1A5 for survival. **(A)** 20 hpi, Mock- or KSHV-infected TIME cells were re-seeded into 24-well plates in triplicate. Cells were treated with replete (REP) media in the presence or absence of 5mM GPNA, a specific inhibitor of SLC1A5, in the presence or absence of 3.5 mM αKG and scanned on the Typhoon at 48 hours post treatment (72 hpi). Glutamine-deprived (-GLUT) controls were included for comparison. Data shown is the average relative fluorescence (YOYO-1 positive cells/SytoGreen24 positive cells) from three independent experiments. Error bars represent the SEM. A *p*-value < 0.01 is denoted by two asterisks. **(B)** TIME cells were transfected with siControl or siSLC1A5. siSLC1A5 treatment leads to an approximately 70% reduction in SLC1A5 expression, determined by qRT-PCR for SLC1A5. Expression was normalized to the housekeeping gene GAPDH. **(C)** Twenty-four hours post transfection of TIME cells with siControl or siSLC1A5, cells were Mock- or KSHV-infected. Upon completion of the infection, cells were treated with replete media containing YOYO-1 to identify dead cells or SytoGreen24 to identify total cell number. 48 hpi (72 hours post transfection), cells were scanned on the Typhoon. Data shown is the average fold change in relative fluorescence of KSHV over mock cells (YOYO-1 positive cells/SytoGreen24 positive cells) from two independent experiments. Error bars represent the SEM.

To determine the role of the glutamine transporter SLC1A5 during latent infection, the SLC1A5 specific inhibitor L-γ-Glutamyl-*p*-nitroanilide (GPNA) was used [29]. Mock- and KSHV-infected TIME cells were re-seeded at 24 hpi and overlaid with replete media or replete media treated with 5mM GPNA. YOYO-1 or SytoGreen24 were added to compare the relative florescence of dead cells and the relative fluorescence of total cells, respectively, at 48 hours post treatment. These experiments were conducted using the Typhoon 9400 variable mode imager to measure relative fluorescence of all samples. GPNA treatment leads to increased cell death only in KSHV-infected cells but not their mock counterparts (Fig 6A). Importantly, when supplemented with 3.5 mM αKG, cell death induced by GPNA treatment of KSHV-infected endothelial cells was rescued to KSHV replete control treatment levels, indicating that the drug-induced cell death was due to the requirement of glutamine metabolism via glutaminolysis and not off-target effects.

To further confirm the drug studies, a validated siRNA set directed to SLC1A5 was used to knockdown SLC1A5 expression[18]. SLC1A5 expression was reduced by approximately 70% in TIME cells transfected with a mix of four siRNAs specific for SLC1A5 (siSLC1A5), as compared to cells transfected with a scrambled non-target control (siControl) (Fig 6B). Twenty-four hours post transfection with the SLC1A5 or control siRNA, cells were either mock- or KSHV-infected and subsequently provided replete media containing YOYO-1 for cell death or SytoGreen24 to identify all cells. Plates were scanned at 48 hours post treatment (72 hpi) for relative fluorescence on the Typhoon 9400 variable mode imager. Minimal cell death was observed in both mock- and KSHV-infected cells treated with siControl. KSHV-infected cells, but not mock-infected cells, transfected with the siSLC1A5 show an increase in cell death. The fold change in relative fluorescence for cell death of KSHV-infected cells over mock-infected cells is increased in cells transfected with siSLC1A5 compared to cells transfected with siControl (Fig 6B). Together, these data support that KSHV-infected endothelial cells rely on the expression of the glutamine transporter SLC1A5 for survival.

### MondoA Regulation of Glutaminolysis Is Required for Survival of Endothelial Cells Latently Infected with KSHV

**Fig 7:**
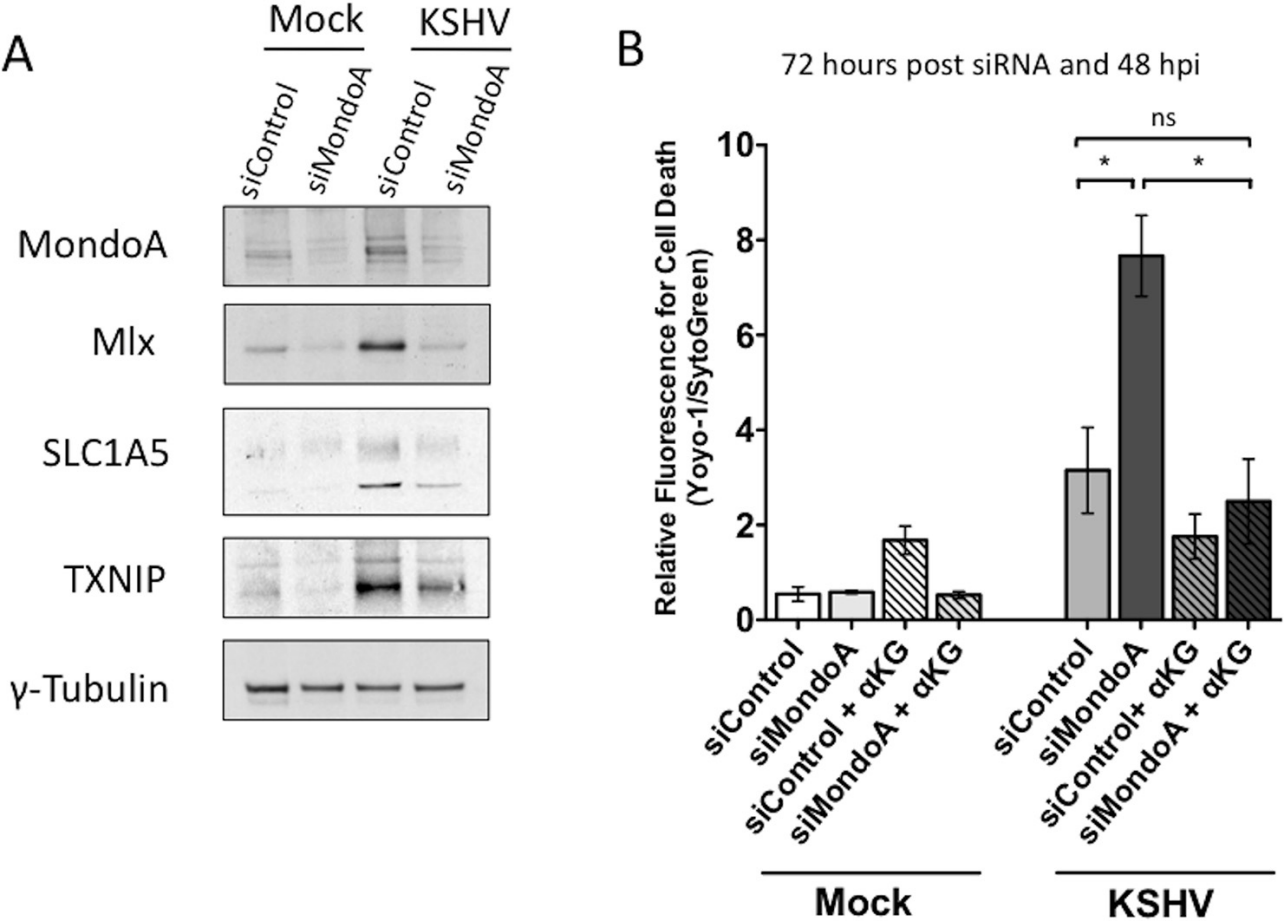
MondoA regulation of glutaminolysis is required for the survival of endothelial cells latently infected with KSHV. **(A)** TIME cells were transfected with siControl or siMondoA and then Mock- or KSHV-infected 24 hours post transfection. Whole-cell lysates were harvested at 48 hpi. Lysates were subjected to immunoblot analysis for MondoA, Mlx, SLC1A5 and TXNIP. KSHV infection elevates levels of all four proteins and loss of MondoA reduces the observed increase in protein expression. The standard γ-tubulin was included as a loading control. **(B)** Twenty-four hours post transfection of TIME cells with siControl or siMondoA, cells were Mock- or KSHV-infected and overlaid with replete media in the presence or absence of αKG. YOYO-1 or SytoGreen was added to the media to monitor relative fluorescence for cell death or total cells, respectively. Forty-eight hpi (72 hours post transfection), cells were scanned on the Typhoon. Data shown is the average fold change in relative fluorescence of KSHV over mock cells (YOYO-1 positive cells/SytoGreen24 positive cells) from two independent experiments. Error bars represent the SEM. A *p* < 0.05 is denoted by one asterisk and “ns” is shown where two averages are not significantly different.

SLC1A5 is directly regulated by the nutrient-sensing Myc extended network member MondoA in many human cancer cells[18]. To determine if MondoA controls SLC1A5 expression during latent KSHV infection of endothelial cells, we examined the expression of SLC1A5 upon siRNA knockdown of MondoA in mock- and KSHV-infected endothelial cells. MondoA protein expression was significantly reduced in both mock and KSHV-infected TIME cells transfected with a mix of four siRNAs specific for MondoA (siMondoA), as compared to cells transfected with a scrambled non-target control (siControl) (Fig 7A). While SLC1A5 protein levels are elevated by KSHV infection, loss of MondoA results in a reduction in detected SLC1A5 in all samples. Additionally, protein levels of Mlx, a co-stabilized MondoA binding partner[18], and TXNIP, a known downstream target of MondoA/Mlx regulation, are also reduced upon loss of MondoA. These data support the hypothesis that MondoA is directly regulating SLC1A5, the major glutamine transporter, during latent KSHV infection of endothelial cells.

To determine if MondoA is required for endothelial cell survival during latent KSHV infection, we examined cell death in the presence of control siRNA or siRNA directed against MondoA. As shown in Fig 7B, only KSHV-infected endothelial cells in the absence of MondoA show a significant increase in cell death at 48 hpi, indicating that MondoA is indeed required for the survival of latently infected cells. Importantly, this significant increase in cell death is fully rescued upon supplementation with αKG, indicating that the cell death that occurs in KSHV-infected cells where MondoA is knocked down is due to a loss of TCA cycle intermediates and not an unrelated function of MondoA.

## Discussion

Transformed cells were first described as ‘glutamine addicted’ in the 1950’s[15]. It is now well established that glutamine, the most abundant amino acid in plasma, is ‘conditionally essential’ for cancer cell growth and survival[13]. More recent evidence shows that lytically replicating viruses orchestrate specific cellular metabolic modifications to support the unique requirements for their viral replication[19, 20, 30–32]. We demonstrate that latent infection with KSHV, an oncogenic virus, induces glutaminolysis in endothelial cells. In addition to showing that latent KSHV infection enhances glutamine uptake during infection, we have shown that a significant percentage of latently infected endothelial cells become glutamine addicted, and that glutaminolysis is required for the survival of these cells. Deprivation of glutamine in both TIME cells and 1°hDMVECs leads to significant increases in apoptosis unless they are supplemented with TCA cycle intermediates.

**Fig 8:**
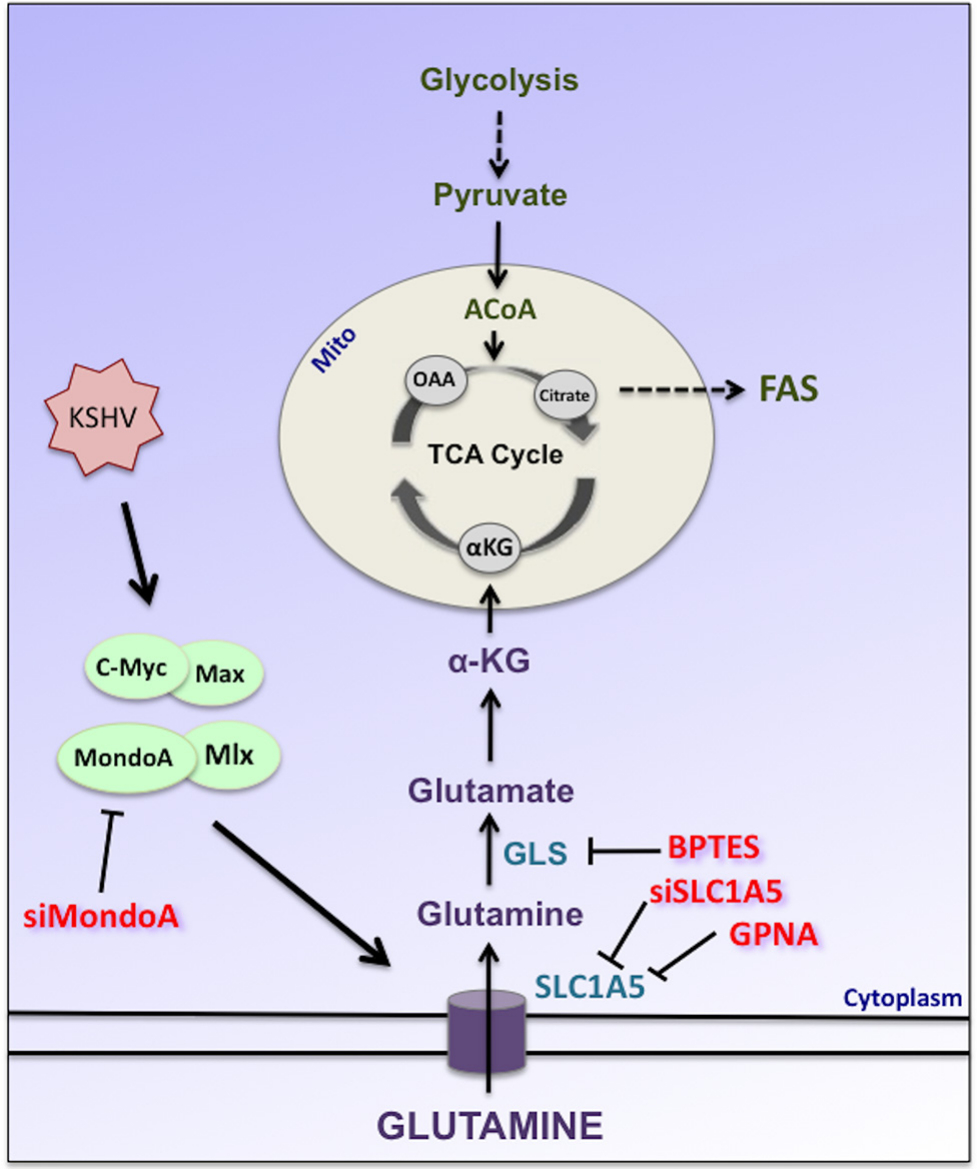
Schematic of glutamine metabolism via glutaminolysis during KSHV infection of endothelial cells. Latent KSHV infection induces and requires the Myc/Max and MondoA/Mlx heterodimers leading to the induction of the glutamine transporter SLC1A5 during latent KSHV infection. Upon entering the cell, glutamine is deaminated twice to form αKG. αKG can enter the TCA cycle where it can be utilized to support bioenergetics and the metabolism of biosynthetic precursors. GPNA or siSLC1A5 treatment was used to specifically inhibit glutamine transport via SLC1A5. BPTES is a specific inhibitor of glutaminase (GLS), the first enzyme of glutaminolysis. siMondoA treatment was used to specifically inhibit MondoA-mediated activation of glutaminolysis.

Glutaminolysis is an important anaplerotic reaction that produces αKG, which can enter the TCA cycle (Fig 8). TCA cycle intermediates support the production of both bioenergetic and biosynthetic precursors; therefore, glutamine is potentially required for a variety of downstream cellular processes including ATP and NADPH production and fatty acid synthesis[33]. There is substantial evidence in cancer biology that glutamine metabolism is required to replenish the TCA cycle when glucose is being metabolized to lactic acid as part of the Warburg effect[13]. Previous research from our lab has shown that induction of the Warburg effect is required for the survival of endothelial cells during latent KSHV infection. Therefore, we were interested in the role glutamine metabolism may play in KSHV-infected endothelial cells. Human cytomegalovirus and vaccinia virus require glutamine to support the TCA cycle for maximal virus replication and media supplemented with TCA cycle intermediates, such as αKG or pyruvate, rescued replication in the absence of glutamine[19, 20]. Our data supports that glutamine is a vital carbon source during latent infection with KSHV.

A recent study reported an increase in glutamate secretion during latent KSHV infection[25]. Glutamate is produced intracellularly through the deamination of glutamine (Fig 8). When glutamate secretion was inhibited, cell proliferation was reduced; however, apoptosis was not reported upon treatment with glutamate secretion inhibitors. Therefore, the increase in glutamine uptake that we observe during latent KSHV infection could be supporting the pleiotropic role of glutamine during infection to support multiple cellular processes, including anaplerosis to support the TCA cycle as well as signaling to the extracellular environment.

We demonstrate that the glutamine transporter SLC1A5 is upregulated during latent KSHV infection of endothelial cells, and that specifically the latently infected cells are dependent upon SLC1A5 for survival. This was of specific interest because previous studies have shown that oncogenic c-Myc, or N-Myc, induces increased expression of the glutamine transporter SLC1A5, and dependency upon it for survival in Myc-activated cells[26]. Multiple studies have reported that c-Myc is upregulated during KSHV infection[25, 34]. We observed an upregulation in c-Myc during infection of endothelial cells, but also identified a significant upregulation in the related proteins MondoA and Mlx. These proteins are a part of the expanded Myc network, known as the Max/Mlx network. MondoA and Mlx form an important glucose-responsive heterodimer that participates in regulating cellular metabolism, specifically glucose, lipid and glutamine metabolism in collaboration with c-Myc or N-Myc. It was recently described that both Myc overexpression and MondoA expression are required to induce the expression of glutamine transporters, including SLC1A5, as well as induce glutaminolysis[18]. We find that MondoA regulation is required for the survival of latently infected endothelial cells and that supplementation with αKG, the immediate downstream intermediate of glutaminolysis and TCA cycle metabolite, promotes cell survival, similarly to our findings upon glutamine deprivation. This is the first evidence of the requirement for MondoA metabolic regulation during human viral infection.

While we have delineated the cellular mechanism of KSHV-induced glutamine addiction, the latent viral gene or set of genes sufficient to induce the MondoA-mediated metabolic switch to glutamine addiction has not yet been determined. Previous research has identified that the latent KSHV protein LANA collaborates with Myc to stabilize and activate the transcriptional regulator during infection[34]. However, this story may be more complicated. It was recently shown that expression of the latent KSHV miRNA cluster is sufficient to induce glucose uptake and glycolysis[11]. If alterations in glucose and glutamine metabolism are interconnected, such as a requirement for glucose to activate MondoA/Mlx, it is likely that multiple viral genes are involved and more work is needed to identify which latent factors are necessary to activate the overall metabolic signature that is required during latent KSHV infection of endothelial cells.

Several major metabolic switches are required during latent KSHV infection; however, the question remains whether induction of cancer cell metabolism is pre-adapting cells for a cancer microenvironment or if these alterations are helping drive oncogenesis when cells are placed in the correct microenvironment. Our findings are in agreement with metabolic signatures described by many cancer studies, which would be predicted if latent KSHV infection is indeed predisposing cells for oncogenesis. However, these models are not necessarily mutually exclusive. Comparing induced metabolic phenotypes, such as the Warburg effect and glutamine addiction in a viral system, where we can include mock controls, provides a unique model to identify the initial drivers of oncogenesis as well as characterize the suitable microenvironment established. Glutamine addiction may be induced early in oncogenesis, yet also be a characteristic of long-term tumor maintenance. Drug inhibitors specifically targeting glutamine-addicted cells could also provide novel therapeutic treatments to specifically target endothelial cells latently infected with KSHV.

## Materials and Methods

### Cells and Viruses

Tert-Immortalized Microvascular Endothelial (TIME) cells [35] and primary human dermal microvascular endothelial cells (1° hDMVECs) (Lonza, MD) were maintained as monolayer cultures in EBM-2 media (Lonza or Cellgro) or EndoGrow (Millipore) supplemented with a bullet kit containing 5% FBS, vascular endothelial growth factor, basic fibroblast growth factor, insulin-like growth factor 3, epidermal growth, and hydrocortisone. Millipore EndoGrow media, supplemented with dialyzed FBS (depleted of small molecules including glucose and glutamine) was used for all experiments that compare replete (4 mM glutamine) and glutamine-free media. KSHV inoculum from induced BCBL-1 cells was titered and used to infect TIME cells or 1° hDMVECs as previously described[36]. Infections were performed in serum-free EBM-2 media and subsequently overlaid with complete EBM-2 media. Infection rates were assessed for each experiment by immunofluorescence and only experiments where greater than 85% of the cells expressed LANA, a latent marker, and less than 1% of the cells expressed ORF59, a lytic marker, were used. In a subset of the siRNA transfection experiments where larger quantities of siRNA were used, there was a slight increase in the cells expressing ORF59, but this always occurred in both the control and gene specific siRNA transfections and did not alter the results of the experiments.

### Reagents and Antibodies

YOYO-1 and SytoGreen24 were diluted in DMSO and used at a final concentration of 100 nM and 50 nM respectively (Life Technologies). Dimethyl-α-ketoglutarate (alpha-ketoglutarate) and pyruvate were purchased from Sigma and used at 3.5 mM and 8 mM respectively. Bis-2-(5-phenylacetamido-1,3,4-thiadiazol-2-yl)ethyl sulfide, or BPTES (Sigma) was solubilized in DMSO, subsequently diluted in methanol and used at a final concentration of 2.5 μM. QVD-OPH (SMBiochemicals) and was dissolved in DMSO and used at a final concentration of 20 μM. L-γ-Glutamyl-*p*-nitroanilide (GPNA) (Sigma), was prepared in DMSO in a 1 M stock solution and used at a final concentration of 5mM.

### Glutamine Uptake Assay

Twenty-four hours post mock- or KSHV-infection; TIME cells were re-seeded into 12-well plates at equal numbers in triplicate. At 96 hpi, cells were overlaid with serum-free media for 2 hours. Cells were then washed three times with PBS before the addition of 1mL of serum-free media containing 0.5μCi (10 pmol) of [^3^H]-L-glutamine (Perkin Elmer #NET551). Cells were incubated for 10 minutes at 37°C. Following incubation, the medium was removed and each well was washed twice with 1mL of ice-cold DPBS and 200μL of lysis buffer (1% SDS in PBS) was added to each well and incubated at room temperature with occasional agitation for 5 minutes. Lysates were transferred to microcentrifuge tubes and mixed by vortexing. 150μL of each lysate was transferred to a vial containing 4mL of Biofluor Plus scintillation fluid (Perkin Elmer). Each vial was mixed by vortexing and counted in a Beckman LS6500 liquid scintillation counter. The remaining lysate was quantified by BCA Protein Assay Reagent Kit (Pierce) for normalization.

### Glutamine Starvation, BPTES and GPNA Treatment Studies

At 20 hpi, mock- and KSHV-infected TIME cells were re-seeded into 24-well plates. At 24 hpi, cells treated with Replete (4 mM glutamine), glutamine-free media or replete media with 2.5 μM BPTES in triplicate. Of note, no changes in latent or lytic infection rates were observed after glutamine starvation. YOYO-1, to identify dead cells, or SytoGreen24, to mark all cell nuclei, were added at this step. For rescue studies, supplementation with 3.5 mM αKG, 8 mM pyruvate or 20 μM QVD were added at this step. Plates were then placed on the IncuCyte (Essen Biosciences), a live-cell phase-contrast and fluorescent imaging system and recorded for cell death and total cell number for 48 hours (24 hpi through 72 hpi). GPNA experiments were prepared according to the same protocol, but scanned on the Typhoon 9400 variable mode imager (GE Healthcare) and analyzed with ImageJ software for relative fluorescence at 48 hours post treatment. Apoptosis experiments conducted with the apoptosis marker, Caspase-3/7 substrate, were prepared according to the same protocol, but the Caspase-3/7 Cell Event reagent was added, plates were scanned at 48 hours post treatment on the Typhoon 9400 and ImageJ software and normalized to relative florescence for Styogreen24. Primary hDMVEC experiments were re-seeded into 24-well plates and at 48 or 72 hpi were treated with Replete (4 mM glutamine) or glutamine-free media and 48 hours post treatment (96 or 120 hpi) were scanned on the Typhoon 9400 variable mode imager (GE Healthcare) and analyzed with ImageJ software for relative fluorescence.

### Western Blot Analysis

All cells were lysed in RIPA and protein was quantified using BCA Assay (Pierce). 30–50ug were subjected to SDS-PAGE in 1xMES Buffer (Life Technologies) on a 4–12% NuPAGE Bis-Tris Gel (Life Technologies) then transferred to 0.2um nitrocellulose membrane (Bio-Rad). The membranes were blocked in 5% Non-Fat Dry Milk in TBS with 0.1% Tween (TBST) for at least an hour then probed with the indicated primary antibodies diluted in 5% milk in TBST for 2 hours at RT, or overnight at 4C (anti-c-Myc (Abcam), anti-Max (Santa Cruz Biotechnology), anti-MondoA (Proteintech), anti-Mlx (Santa Cruz Biotechnology), anti-SCL1A5 (Cell Signaling) and anti-TXNIP (MBL, JY1). Blots were washed 3 times in TBST, then probed with HRP-conjugated secondary antibody (Cell Signaling) diluted in 5% milk in TBST for 1 hour at RT. Blots were washed 3 times in TBST, then subjected to chemiluminescence and exposed to blue autoradiography film (GeneMate) and processed in an autoprocessor.

### Quantitative Real-Time Reverse Transcription PCR (qRT-PCR)

Total RNA was isolated from TIME cells 72 hours post siRNA transfection using the Nucleospin RNA II Kit (Macherey-Nagel). Two-step quantitative real-time reverse transcription PCR (BioRad) was used to measure expression levels of SLC1A5 and the housekeeping gene GAPDH. iScript Reverse Transcription Supermix and SsoAdvanced SYBR Green Supermix (BioRad) were used according to manufacturer’s protocols. The primers used were: SLC1A5-F ‘5-TTATCCGCTTCTTCAACTCCTT-3’, SLC1A5-R ‘5-ACATCCTCCATCTCCACGAT-3’, or GAPDH-F: ‘5-GGACTCATGACCACAGTCCA-3’, GAPDH-R ‘5-CCAGTAGAGGCAGGGATGAT-3’. Relative levels of SLC1A5 mRNA were normalized by the delta threshold cycle method to the abundance of GAPDH mRNA.

### siRNA Transfection and Cell Survival

A set of four siRNAs specific to the glutamine transporter SLC1A5 (siSLC1A5) and MondoA (siMondoA) were purchased (Qiagen, Flexitube GeneSolution #GS6510 and #GS22877 respectively). A negative-control siRNA (siControl) was designed and synthesized by Ambion. TIME cells were transfected with siRNA at a final concentration of 200 nM, using the Amaxa Nucleofector Kit by Lonza according to the manufacturer’s protocol. At 24 hours post transfection, cells were mock- or KSHV-infected. Upon completion of the infection, cells were washed and treated with Replete media containing YOYO-1 or SytoGreen24. Relative fluorescence was measured 48 hours post treatment using a Typhoon 9400 variable mode imager (GE Healthcare) and ImageJ software.

## References

[1] Dourmishev LA, Dourmishev AL, Schwartz RA, Lukac DM, Palmeri D. Molecular Genetics of Kaposi ' s Sarcoma-Associated Herpesvirus (Human Herpesvirus 8) Epidemiology and Pathogenesis. Microbiology and Molecular Biology Reviews. 2003;67(2):175–212. 12794189 10.1128/MMBR.67.2.175-212.2003 PMC156467

[2] Mesri Ea, Cesarman E, Boshoff C. Kaposi's sarcoma and its associated herpesvirus. Nature reviews Cancer. 2010;10(10):707–19. 10.1038/nrc2888 20865011 PMC4721662

[3] Wabinga HR, Parkin DM, Wabwire-Mangen F, Mugerwa JW. Cancer in Kampala, Uganda, in 1989–91: changes in incidence in the era of AIDS. International journal of cancer Journal international du cancer. 1993;54(1):26–36. 8478145 10.1002/ijc.2910540106

[4] Chokunonga E, Levy LM, Bassett MT, Mauchaza BG, Thomas DB, Parkin DM. Cancer incidence in the African population of Harare, Zimbabwe: second results from the cancer registry 1993–1995. International journal of cancer Journal international du cancer. 2000;85(1):54–9. 10585583 10.1002/(sici)1097-0215(20000101)85:1<54::aid-ijc10>3.0.co;2-d

[5] Lagunoff M, Bechtel J, Venetsanakos E, Roy A-m, Abbey N, Herndier B, et al De Novo Infection and Serial Transmission of Kaposi' s Sarcoma-Associated Herpesvirus in Cultured Endothelial Cells. Journal of Virology. 2002;76(5):2440–8. 11836422 10.1128/jvi.76.5.2440-2448.2002 PMC153827

[6] Zhong W, Wang H, Herndier B, Ganem D. Restricted expression of Kaposi sarcoma-associated herpesvirus (human herpesvirus 8) genes in Kaposi sarcoma. Proc Natl Acad Sci U S A. 1996;93(13):6641–6. 8692871 10.1073/pnas.93.13.6641 PMC39079

[7] Delgado T, Carroll PA, Punjabi AS, Margineantu D, Hockenbery DM. Induction of the Warburg effect by Kaposi’ s sarcoma herpesvirus is required for the maintenance of latently infected endothelial cells. Proceedings of the National Academy of Sciences. 2010;107(23):10696–701. www.pnas.org/cgi/doi/10.1073/pnas.1004882107. 10.1073/pnas.1004882107 PMC2890792 20498071

[8] Delgado T, Sanchez EL, Camarda R, Lagunoff M. Global metabolic profiling of infection by an oncogenic virus: KSHV induces and requires lipogenesis for survival of latent infection. PLoS pathogens. 2012;8(8):e1002866–e. 10.1371/journal.ppat.1002866 22916018 PMC3420960

[9] Bhatt AP, Jacobs SR, Freemerman AJ, Makowski L, Rathmell JC, Dittmer DP, et al Dysregulation of fatty acid synthesis and glycolysis in non-Hodgkin lymphoma. Proceedings of the National Academy of Sciences of the United States of America. 2012;109(29):11818–23. 10.1073/pnas.1205995109 22752304 PMC3406848

[10] Hsu PP, Sabatini DM. Cancer cell metabolism: Warburg and beyond. Cell. 2008;134(5):703–7. 10.1016/j.cell.2008.08.021 18775299

[11] Yogev O, Lagos D, Enver T, Boshoff C. Kaposi's sarcoma herpesvirus microRNAs induce metabolic transformation of infected cells. PLoS Pathog. 2014;10(9):e1004400 10.1371/journal.ppat.1004400 25255370 PMC4177984

[12] Vander Heiden MG, Cantley LC, Thompson CB. Understanding the Warburg effect: the metabolic requirements of cell proliferation. Science (New York, NY). 2009;324(5930):1029–33. 10.1126/science.1160809 PMC2849637 19460998

[13] DeBerardinis RJ, Cheng T. Q's next: the diverse functions of glutamine in metabolism, cell biology and cancer. Oncogene. 2010;29(3):313–24. 10.1038/onc.2009.358 19881548 PMC2809806

[14] Hensley CT, Wasti AT, DeBerardinis RJ. Glutamine and cancer: cell biology, physiology, and clinical opportunities. J Clin Invest. 2013;123(9):3678–84. 10.1172/JCI69600 23999442 PMC3754270

[15] Eagle BYH. Nutrition Needs of Mammalian Cells in Tissue Culture. Science. 1955;122(3168):501–4. 13255879 10.1126/science.122.3168.501

[16] Wise DR, DeBerardinis RJ, Mancuso A, Sayed N, Zhang X-Y, Pfeiffer HK, et al Myc regulates a transcriptional program that stimulates mitochondrial glutaminolysis and leads to glutamine addiction. Proceedings of the National Academy of Sciences. 2008;105(48):18782–7. 10.1073/pnas.0810199105 PMC2596212 19033189

[17] Yuneva M, Zamboni N, Oefner P, Sachidanandam R, Lazebnik Y. Deficiency in glutamine but not glucose induces MYC-dependent apoptosis in human cells. The Journal of cell biology. 2007;178(1):93–105. 17606868 10.1083/jcb.200703099 PMC2064426

[18] Carroll PA, Diolaiti D., McFerrin L., Gu H., Djukovic D., Du J., Cheng P.F., Anderson S., Ulrich M., Hurley J.B., Raftery D., Ayer D.E., Eisenman R.N.. Deregulated Myc Requires MondoA/Mlx for Metabolic Reprogramming and Tumorigenesis. Cancer Cell. 2015;27:1–15. 25640402 10.1016/j.ccell.2014.11.024 PMC4326605

[19] Chambers JW, Maguire TG, Alwine JC. Glutamine metabolism is essential for human cytomegalovirus infection. Journal of virology. 2010;84(4):1867–73. 10.1128/JVI.02123-09 19939921 PMC2812398

[20] Fontaine KA, Camarda R, Lagunoff M. Vaccinia virus requires glutamine but not glucose for efficient replication. J Virol. 2014;88(8):4366–74. 10.1128/JVI.03134-13 24501408 PMC3993723

[21] Eagle BYH, Habel AK. The Importance of Glucose and Glutamine for the Elaboration of Poliomyditis Virus by HeLa Cell. Journal of Experimental Medicine. 1956;104(2):271–87. 13345971 10.1084/jem.104.2.271 PMC2136654

[22] Daye D, Wellen KE. Metabolic reprogramming in cancer: unraveling the role of glutamine in tumorigenesis. Semin Cell Dev Biol. 2012;23(4):362–9. 10.1016/j.semcdb.2012.02.002 22349059

[23] Dang CV. MYC, metabolism, cell growth, and tumorigenesis. Cold Spring Harbor perspectives in medicine. 2013;3(8). 10.1101/cshperspect.a014217 PMC3721271 23906881

[24] Li X, Chen S, Feng J, Deng H, Sun R. Myc is required for the maintenance of Kaposi's sarcoma-associated herpesvirus latency. J Virol. 2010;84(17):8945–8. 10.1128/JVI.00244-10 20573831 PMC2919007

[25] Valiya Veettil M, Dutta D, Bottero V, Bandyopadhyay C, Gjyshi O, Sharma-Walia N, et al Glutamate secretion and metabotropic glutamate receptor 1 expression during Kaposi's sarcoma-associated herpesvirus infection promotes cell proliferation. PLoS Pathog. 2014;10(10):e1004389 10.1371/journal.ppat.1004389 25299066 PMC4192595

[26] Wang R, Dillon CP, Shi LZ, Milasta S, Carter R, Finkelstein D, et al The transcription factor Myc controls metabolic reprogramming upon T lymphocyte activation. Immunity. 2011;35(6):871–82. 10.1016/j.immuni.2011.09.021 22195744 PMC3248798

[27] Diolaiti D, McFerrin L, Carroll PA, Eisenman RN. Functional interactions among members of the MAX and MLX transcriptional network during oncogenesis. Biochim Biophys Acta. 2014. 10.1016/j.bbagrm.2014.05.016 PMC4241192 24857747

[28] Pochini L, Scalise M, Galluccio M, Indiveri C. Membrane transporters for the special amino acid glutamine: structure/function relationships and relevance to human health. Frontiers in chemistry. 2014;2:61 10.3389/fchem.2014.00061 25157349 PMC4127817

[29] Ren P, Yue M, Xiao D, Xiu R, Gan L, Liu H, et al ATF4 and N-Myc coordinate glutamine metabolism in MYCN-amplified neuroblastoma cells through ASCT2 activation. The Journal of pathology. 2015;235(1):90–100. 10.1002/path.4429 25142020

[30] Vastag L, Koyuncu E, Grady SL, Shenk TE, Rabinowitz JD. Divergent effects of human cytomegalovirus and herpes simplex virus-1 on cellular metabolism. PLoS pathogens. 2011;7(7):e1002124–e. 10.1371/journal.ppat.1002124 21779165 PMC3136460

[31] Fontaine KA, Sanchez EL, Camarda R, Lagunoff M. Dengue Virus Induces and Requires Glycolysis for Optimal Replication. J Virol. 2014. 10.1128/JVI.02309-14 PMC4338897 25505078

[32] Munger J, Bennett BD, Parikh A, Feng X-J, McArdle J, Rabitz Ha, et al Systems-level metabolic flux profiling identifies fatty acid synthesis as a target for antiviral therapy. Nature biotechnology. 2008;26(10):1179–86. 10.1038/nbt.1500 18820684 PMC2825756

[33] Deberardinis RJ, Sayed N, Ditsworth D, Thompson CB. Brick by brick: metabolism and tumor cell growth. Current opinion in genetics & development. 2008;18(1):54–61. 18387799 10.1016/j.gde.2008.02.003 PMC2476215

[34] Liu J, Martin HJ, Liao G, Hayward SD. The Kaposi's sarcoma-associated herpesvirus LANA protein stabilizes and activates c-Myc. J Virol. 2007;81(19):10451–9. 17634226 10.1128/JVI.00804-07 PMC2045471

[35] Venetsanakos E, Mirza A, Fanton C, Romanov SR, Tlsty T, McMahon M. Induction of tubulogenesis in telomerase-immortalized human microvascular endothelial cells by glioblastoma cells. Experimental cell research. 2002;273(1):21–33. 11795943 10.1006/excr.2001.5424

[36] Punjabi AS, Carroll PA, Chen L, Lagunoff M. Persistent activation of STAT3 by latent Kaposi's sarcoma-associated herpesvirus infection of endothelial cells. J Virol. 2007;81(5):2449–58. 17151100 10.1128/JVI.01769-06 PMC1865938

